# Novel Population Pharmacokinetic Approach to Explain the Differences between Cystic Fibrosis Patients and Healthy Volunteers via Protein Binding

**DOI:** 10.3390/pharmaceutics11060286

**Published:** 2019-06-18

**Authors:** Nirav R. Shah, Jürgen B. Bulitta, Martina Kinzig, Cornelia B. Landersdorfer, Yuanyuan Jiao, Dhruvitkumar S. Sutaria, Xun Tao, Rainer Höhl, Ulrike Holzgrabe, Frieder Kees, Ulrich Stephan, Fritz Sörgel

**Affiliations:** 1 Department of Pharmacotherapy and Translational Research, College of Pharmacy, University of Florida, Orlando, FL 32827, USA; shah.nirav@cop.ufl.edu (N.R.S.); yyjiao@cop.ufl.edu (Y.J.); DSutaria@cop.ufl.edu (D.S.S.); tealingsxun@ufl.edu (X.T.); 2 IBMP—Institute for Biomedical and Pharmaceutical Research, Nürnberg-Heroldsberg 90562, Germany; Martina.Kinzig@gtf-online.de; 3 Drug Delivery, Disposition and Dynamics, Monash Institute of Pharmaceutical Sciences, Monash University, Parkville, VIC 3052, Australia; cornelia.landersdorfer@monash.edu; 4 Institute of Clinical Hygiene, Medical Microbiology and Infectiology, Klinikum Nürnberg, Paracelsus Medical University, Nürnberg 90419, Germany; rai.hoehl@googlemail.com; 5 Institute for Pharmacy and Food Chemistry, University of Würzburg, Würzburg 97074, Germany; ulrike.holzgrabe@uni-wuerzburg.de; 6 Department of Pharmacology, University of Regensburg, Regensburg 93053, Germany; frieder.kees@chemie.uni-regensburg.de; 7 Department of Pharmacology, University of Duisburg, Essen 47057, Germany

**Keywords:** cystic fibrosis patients, healthy volunteers, cefotiam, beta-lactam antibiotics, population pharmacokinetics, protein binding, allometric scaling, body size, body composition, S-ADAPT

## Abstract

The pharmacokinetics in patients with cystic fibrosis (CF) has long been thought to differ considerably from that in healthy volunteers. For highly protein bound β-lactams, profound pharmacokinetic differences were observed between comparatively morbid patients with CF and healthy volunteers. These differences could be explained by body weight and body composition for β-lactams with low protein binding. This study aimed to develop a novel population modeling approach to describe the pharmacokinetic differences between both subject groups by estimating protein binding. Eight patients with CF (lean body mass [LBM]: 39.8 ± 5.4kg) and six healthy volunteers (LBM: 53.1 ± 9.5kg) received 1027.5 mg cefotiam intravenously. Plasma concentrations and amounts in urine were simultaneously modelled. Unscaled total clearance and volume of distribution were 3% smaller in patients with CF compared to those in healthy volunteers. After allometric scaling by LBM to account for body size and composition, the remaining pharmacokinetic differences were explained by estimating the unbound fraction of cefotiam in plasma. The latter was fixed to 50% in male and estimated as 54.5% in female healthy volunteers as well as 56.3% in male and 74.4% in female patients with CF. This novel approach holds promise for characterizing the pharmacokinetics in special patient populations with altered protein binding.

## 1. Introduction

The pharmacokinetics (PK) of patients with cystic fibrosis (CF) has been reported to considerably differ from that in healthy volunteers since the 1970s [1,2,3,4]. This was especially true for early studies in patients with CF for β-lactams with high protein binding (such as dicloxacillin and cloxacillin) [5,6]. These early studies compared mostly adolescent and presumably rather morbid patients with CF to adult healthy volunteers and found up to 2.07-fold higher unbound fractions for some β-lactams in plasma of patients with CF (e.g., 11.6 ± 7.7% in patients with CF versus 5.6 ± 1.9% in healthy volunteers for dicloxacillin).

Recent PK studies [7,8,9,10,11] assessed β-lactams with anti-pseudomonal activity; these compounds had low or intermediate protein binding with unbound fractions of 49% or higher. These studies employed population PK modeling to account for the differences in body size and body composition via allometric scaling based on lean body mass (LBM). This approach explained most of the differences in clearance and volume of distribution between both subject groups [3]. However, for aztreonam, the most highly bound β-lactam in these newer studies [9], the unbound fraction in serum was approximately 19% higher in patients with CF compared to that in healthy volunteers. When calculated based on total serum concentrations, the aztreonam clearance was 31% higher in patients with CF compared to that in healthy volunteers who were matched in terms of body size and body composition. After accounting for the difference in protein binding, unbound clearance was only 10% higher in patients with CF [9]. This was in good agreement with the PK differences for other β-lactams with low protein binding [7,8,10,11] and with generally similar (or only slightly higher) renal function in patients with CF compared to that in healthy volunteers [1,12,13,14].

Cefotiam is a β-lactam with intermediate protein binding ranging from 40% to 62% in different studies [15,16,17,18]. Cefotiam has never been studied in patients with CF and only one small study assessed its population PK in patients with intra-abdominal infections [19]. Population modeling allows one to simultaneously estimate the population mean PK parameters and their between subject variability (BSV). This approach can further describe differences in body size and body composition via allometric scaling [20] based on total body weight (WT) or LBM, for example. Moreover, this methodology is the foundation of Monte Carlo simulations which predict the probability of attaining a pharmacokinetic/pharmacodynamic (PK/PD) target which correlates with efficacy in mice and patients. Specifically for β-lactam antibiotics, the duration during which the non-protein bound plasma concentration exceeds the minimal inhibitory concentration (*f*T_>MIC_) was found to best predict bacterial killing at 24 h in both mouse infection models and clinical outcomes in patients [21,22,23,24].

This study aimed to develop a novel population modeling approach for characterizing the PK differences between patients with CF and healthy volunteers by estimating protein binding in both subject groups. Secondly, we sought to predict the probability of target attainment (PTA) for cefotiam in both subject groups. We accounted for the differences in body size and body composition via allometric scaling by LBM. When clearances and volumes of distribution were calculated based on total drug concentrations, these PK parameter estimates were 11% to 38% larger in patients with CF compared to those in healthy volunteers. After allometric scaling of PK parameters by LBM, the remaining differences in clearance and volume of distribution could be explained by a higher modelled unbound fraction for cefotiam in the plasma of patients with CF compared to that of healthy volunteers. This novel approach holds promise to characterize PK differences for drugs with moderate or high protein binding which may be affected by pathophysiological alterations in special patient populations.

## 2. Materials and Methods

### 2.1. Subjects

A total of 14 Caucasian subjects (eight patients with CF and six healthy volunteers) participated in the study after they had given their written informed consent. For one patient with CF aged 17 years, written informed consent was obtained from the legal representative. The general clinical procedures in the present study were the same as those described previously [7,8,11]. The study protocol had been approved by the ethics committee of the University Hospital Essen under the title “Pharmakokinetik von Antibiotika bei Mukoviszidose-Patienten und gesunden Probanden” (approved on 29 August, 1984) and the study was performed in concordance with the revised version of the Declaration of Helsinki.

### 2.2. Study Design and Drug Administration

This study was a single dose, single-center, open, parallel group trial. All subjects received 1027.5 mg cefotiam as 3 min intravenous infusion via a BRAUN-Perfusor^®^ (Braun, Melsungen, Germany). The performance of these instruments was assured on a daily basis by weighing defined volumes delivered by the perfusors.

### 2.3. Blood Sampling

All blood samples were drawn from a forearm vein via an intravenous catheter contralateral to the one used for dosing. Blood samples were drawn immediately before the start of the infusion, at the end of the infusion (3 min), as well as at 5, 10, 15, 20, 30, 45, 60, 90 min, and 2, 3, 4, 5, 6, 8, 12 and 24 h post the end of infusion. The samples were cooled in an ice-water bath for 10–15 min before centrifugation. After centrifugation, all of the plasma samples were immediately frozen and stored at −70 °C until analysis.

### 2.4. Urine Collections

Urine was collected to determine the fraction of drug eliminated into urine and to estimate renal clearance. Sampling intervals were pre-dose as well as from the start of infusion (0 h) to 1, 1 to 2, 2 to 3, 3 to 4, 4 to 5, 5 to 6, 6 to 8, 8 to 12, and 12 to 24 h post start of the infusion. After dosing, subjects were asked to drink 200 mL of mineral water or apple juice to support diuresis. Urine samples were collected into individually weighed urine sampling containers and stored at +4 °C during the sampling interval. Thereafter, the amount and pH of the urine were measured and aliquots were immediately frozen and stored at −70 °C until analysis.

### 2.5. Drug Analysis

Cefotiam concentrations in plasma were determined by reversed phase high performance liquid chromatography (HPLC) using a validated assay [25]. In brief, 200 µL of NaH_2_PO_4_ buffer at pH 6.2 was added to 200 µL of each plasma sample. Acetonitrile (400 µL) was used to deproteinize each sample. After centrifugation, 2000 µL of dichloromethane were added for extraction of acetonitrile. From the remaining aqueous phase, 20 to 40 µL were injected into the HPLC system. Urine was centrifuged and diluted 1:10 with 50 mM of sodium phosphate buffer at pH 7.0. A volume of 10 µL of this diluted solution was injected into the HPLC system. The recovery from plasma was 99.7 ± 1.6% at a concentration of 100 mg/L, 99.6 ± 3.3% at 25 mg/L and 94.9 ± 5.9% at 1 mg/L. For comparison, the corresponding recoveries from water were 97.2 ± 3.0%, 94.1 ± 2.6% and 95.3 ± 1.9%, respectively.

A Novapack C18 (5 µm) column was used with a water/acetonitrile mixture at pH 4.7. Cefotiam was detected at 254 nm. A volume of 30–40 mL blood was drawn from one additional subject at three time points with a high, intermediate and low concentration. This subject did not participate in the main part of the PK study. Those blood samples were used as incurred samples (i.e., biological quality controls) that were included in all analytical runs.

### 2.6. Population Pharmacokinetic Analysis

#### 2.6.1. Population Model

We tested one, two and three compartment disposition models and compared models based on the objective function (-1x log-likelihood in S-ADAPT), individual curve fit plots, their predictive performance assessed via visual predictive checks and normalized prediction-distribution errors, as well as other standard diagnostic plots [11,26,27,28]. Visual predictive checks assessed whether the median and the prediction intervals mirrored the central tendency and the variability of the observations.

#### 2.6.2. Modeling Approach

After identifying the most suitable model structure, we evaluated various models to account for body size and body composition. We employed two strategies to describe the remaining differences between patients with CF and healthy volunteers as well as potential differences between males and females. The first approach used disease specific scale factors (FCYF) for clearance and volume of distribution to describe differences between both subject groups as we described previously [7,8,10,11]. As an alternative, we employed a novel strategy to estimate different unbound fractions for cefotiam in plasma for male and female patients with CF and healthy volunteers. The latter approach does not require the FCYF and may be suitable for drugs with intermediate or high protein binding. We considered models where the entire renal clearance (i.e., glomerular filtration and tubular secretion) was affected by the estimated plasma protein binding and alternative models where only glomerular filtration but not tubular secretion was affected by protein binding. We used literature data on albumin concentrations in patients with CF and healthy volunteers to support the estimated differences in protein binding [29] (as described in the Supplement).

#### 2.6.3. Body Size and Composition

We compared five different models for body size and body composition: 1) No size model, 2) linear scaling by WT, 3) allometric scaling by WT [30,31,32], 4) linear scaling by LBM [33,34] and 5) allometric scaling by LBM. We compared the ability of each body size model to describe the differences in the mean PK parameters between both subject groups and to reduce the unexplained BSV. For linear scaling, all exponents were fixed to 1.0. The allometric body size models used a fixed exponent of 1.0 (i.e., linear scaling) for volume of distribution and a fixed exponent of 0.75 (i.e., slightly less than linear scaling) for clearances. The F_Size,V,i_ and F_Size,CL,i_ represent the fractional changes in volume of distribution and clearance for the ith subject (with LBM_i_) standardized to an LBM_STD_ of 53 kg (equivalent to a standard weight of 70 kg).

(1) $$F_{\mathrm{Size},V,i}\;=\;\frac{{\mathrm{LBM}}_{i}}{{\mathrm{LBM}}_{\mathrm{STD}}}$$

(2) $$F_{\mathrm{Size},\mathrm{CL},i}\;=\;{\left(\frac{{\mathrm{LBM}}_{i}}{{\mathrm{LBM}}_{\mathrm{STD}}}\right)}^{0.75}$$

#### 2.6.4. Between-Subject Variability Model

The BSV for clearances and volumes of distribution was described by log-normal distributions. The η_BSV_ was a normally distributed random variable with mean zero and standard deviation BSV. The individual PK parameters were calculated as:

(3) $${\mathrm{CLr}}_{i}\;=\;\mathrm{CLr}\;\cdot \;F_{\mathrm{Size},\mathrm{CL},i}\;\cdot \;{\mathrm{FCYF}}_{\mathrm{CLr}}\;\cdot \;\exp\left(\eta_{\mathrm{BSVCLr},i}\right)$$

The CLr_i_ is the individual renal clearance estimate and η_BSVCLr,i_ is the random deviate of CLr for the ith subject. The population mean renal clearance (CLr) applies to healthy volunteers with a standard body size (e.g., an LBM_STD_ of 53 kg). The disease factor for patients with CF (e.g., FCYF_CLr_) characterizes the ratio of the mean renal clearance for patients with CF divided by that in healthy volunteers. An FCYF_CLr_ of 1.0 means that patients with CF and healthy volunteers of the same body size have identical group estimates for renal clearance. We used disease specific scale factors for renal and nonrenal clearance as well as for volume of distribution at steady-state (FCYF_VSS_).

#### 2.6.5. Observation Model

The residual unidentified variability was described by a combined additive plus proportional residual error model for plasma concentrations. The fractions of dose excreted into urine as unchanged cefotiam were fit using an additive residual error model. Simultaneous fitting of plasma concentrations and the fractions excreted into urine allowed us to estimate both renal and nonrenal clearance.

After scaling by body size and composition, we accounted for potential differences in protein binding to explore additional PK differences between both subject groups. We fixed the unbound fraction of cefotiam in plasma of healthy male volunteers to 50% based on literature data [15,16,17,18] and estimated the unbound fraction in female healthy volunteers as well as male and female patients with CF. The observed plasma concentration of total cefotiam was calculated as the modelled unbound cefotiam concentration divided by the unbound fraction. This approach not only allowed us to estimate potential differences in the unbound fraction between both subject groups and sexes; it also allowed us to explain PK differences between the four groups which could not be described by scaling for body size and composition. When we estimated different unbound fractions for male and female patients with CF and healthy volunteers, all disease specific scale factors FCYF were removed from the model and the resulting unbound renal clearance (CLr_u_) was calculated as:

(4) $${\mathrm{CLr}}_{u,i}\;=\;{\mathrm{CLr}}_{u}\;\cdot \;F_{\mathrm{Size},\mathrm{CL},i}\;\cdot \;\exp\left(\eta_{\mathrm{BSVCLr},u,i}\right)$$

All abbreviations for unbound renal clearance carry the same meaning as those described above for renal clearance based on total drug concentrations.

#### 2.6.6. Estimation and Computation

The importance sampling algorithm (pmethod = 4) in S-ADAPT (version 1.57) [35] was used for all population modelling which was facilitated by the SADAPT-TRAN package [36,37]. Phoenix/WinNonlin Professional (version 8.1.0, Certara L.P., Princeton, NJ, USA) was used for non-compartmental analysis and statistics.

#### 2.6.7. Monte Carlo Simulations

Based on the final population PK model, we performed Monte Carlo simulations to predict the time-course of unbound cefotiam concentrations in the plasma of patients with CF and healthy volunteers. We simulated 4000 virtual subjects for each dosage regimen at a daily dose of 3000 mg cefotiam. Simulations included 3 min infusions of 1000 mg every 8 h, prolonged (3 h) infusions of 1000 mg every 8 h and a continuous infusion of 3000 mg/day (with a 500 mg loading dose at 0 h to rapidly attain a steady-state). The geometric mean LBM used for Monte Carlo simulations was 40 kg for male and female patients with CF, as well as 61 kg for healthy male and 45 kg for healthy female volunteers. The same geometric mean LBM in male and female patients with CF was used during simulations to assess whether the probability of target attainment was affected by sex when using a fixed (i.e., not LBM-adjusted) cefotiam dose. The covariate distribution model used a log-normal distribution with a 15% coefficient of variation for LBM in each subject group.

The time of the unbound cefotiam concentration in plasma above the minimal inhibitory concentration (*f*T_>MIC_) was shown to be the most predictive PK/PD index for cephalosporins [22,23]. The *f*T_>MIC_ was calculated by numerical integration at steady-state using the Berkeley Madonna software (version 8.3.18) [11,38]. The fractions of patients achieving the PK/PD targets for bacteriostasis (40% *f*T_>MIC_) and near-maximal bacterial killing (65% *f*T_>MIC_) at 24 h in mouse infection models were calculated to estimate the probabilities of target attainment as described previously [21,23]. The PK/PD breakpoint was defined and reported as the highest minimal inhibitory concentrations (MIC) with a probability of target attainment of at least 90%. For these Monte Carlo simulations, the unbound fraction for cefotiam in plasma was fixed to 50% for male healthy volunteers [15,16,17,18] and the estimated unbound fractions in the three other subject groups were used during simulations.

## 3. Results

Patients with CF were smaller and leaner than the healthy volunteers in this study (Table 1). While female and male patients with CF had comparable LBM, male healthy volunteers had a 41% larger LBM compared to female healthy volunteers. The average plasma concentration profiles over time were similar between both subject groups, except for higher peak concentrations in patients with CF (Figure S1).

Non-compartmental analysis showed that total, renal and non-renal clearance as well as volume of distribution at steady-state were comparable between patients with CF and healthy volunteers (Table 2). Terminal half-life was 14% shorter in patients with CF. For the latter, total clearance was 38% larger in females compared to males and the same trend was observed for renal and non-renal clearance (Table 2); this was despite male and female patients with CF having similar LBM (Table 1). Of note, these non-compartmental PK parameters (Table 2) did not account for differences in body size and body composition.

The fraction excreted as unchanged cefotiam into urine was similar between both subject groups. Six of eight patients with CF excreted more than 70% of the cefotiam dose into urine, whereas one patient with CF only excreted 47.6%. The population PK analysis was performed with and without the urine data for this patient and removing the urine data of this patient had minimal impact on the reported final population mean PK parameter estimates.

### Population Pharmacokinetic Modeling

#### Structural Model

**Figure 1:**
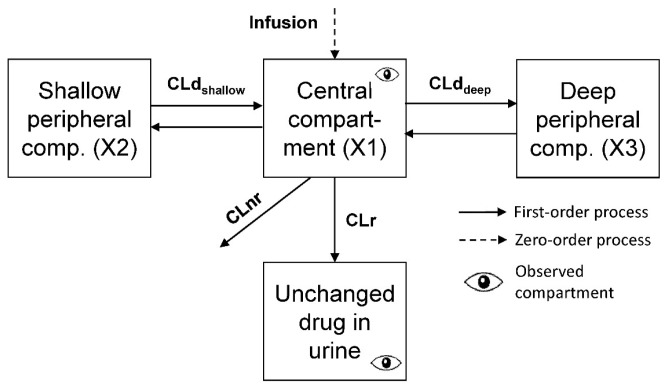
Three-compartment model structure for cefotiam in plasma and urine.

Visual predictive checks and other diagnostic plots suggested that both the two- and three-compartment models adequately described the data. However, the three-compartment model (Figure 1) described the terminal phase better, yielded a significantly better objective function (*p* < 0.001, likelihood ratio test) and was chosen as the final structural model.

#### Body Size Models

In our first approach to account for the differences between patients with CF and healthy volunteers, we introduced the FCYF for clearances and volume of distribution. Without scaling by body size, patients with CF and healthy volunteers had FCYF_CLr_, FCYF_CLnr_ and FCYF_Vss_ (Table 3) close to 1.0 in agreement with the non-compartmental results (Table 2). Linear and allometric scaling by WT as well as linear scaling by LBM yielded approximately 19% to 52% larger clearances and volumes of distribution in patients with CF compared to estimates in healthy volunteers (Table 3). For allometric scaling by LBM, the scale factors were 1.21 for renal and 1.11 for nonrenal clearance, as well as 1.38 for volume of distribution at steady-state. The complete set of population PK parameter estimates for the model with FCYF and allometric scaling by LBM is shown in Table S1.

#### Estimating Unbound Fractions

As a second strategy to explain the differences between patients with CF and healthy volunteers, we estimated different unbound fractions (fu) of cefotiam in plasma. These models did not include any disease specific scale factors (i.e., all FCYF were removed). Allometric scaling by LBM was used to account for body size and body composition and different unbound fractions for males and females were estimated in each subject group (Table 4; unbound fraction in male healthy volunteers was fixed to 50%).

**Figure 2:**
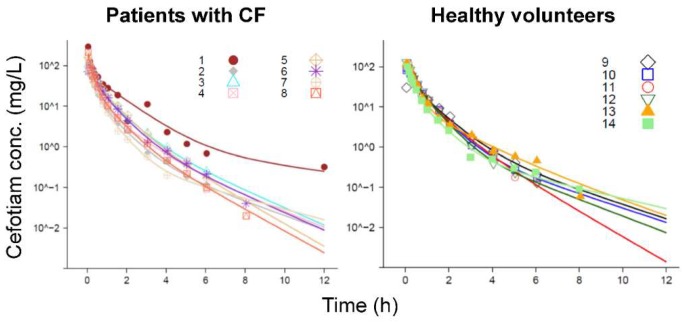
Observed plasma concentrations and individual curve fits (lines) for cefotiam in patients with CF (left) and healthy volunteers (right). The legends show the subject numbers.

**Figure 3:**
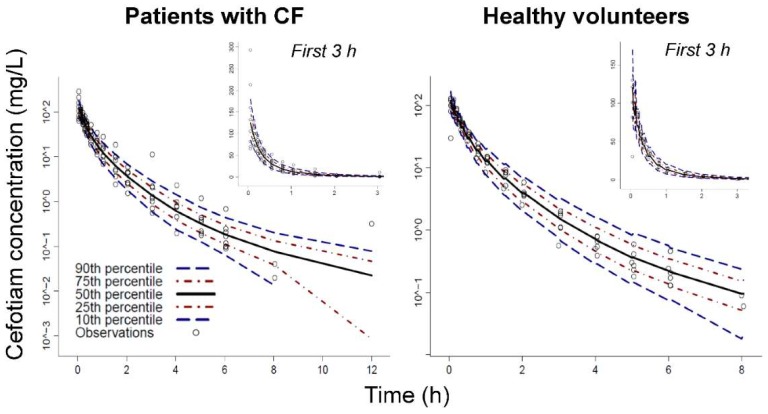
Visual predictive check for cefotiam concentrations in patients with CF (left) and healthy volunteers (right). The plots show the observations (markers), the 50th percentile (i.e., median) of the model predictions (black line) along with the 80% prediction interval [10th to 90th percentile] and the interquartile range [25th to 75th percentile]. Ideally, the median of the observations and of the predictions should superimpose and 10% of the observations should fall outside the 80% prediction interval on either side.

The final model estimated higher unbound fractions in female compared to male patients with CF (females: 0.744 versus males: 0.563) and in female compared to male healthy volunteers (females: 0.545 versus males: 0.50 [fixed]; Table 4). This model yielded excellent individual curve fits (Figure 2) and good predictive performance (Figure 3) in both subject groups. Standard diagnostic plots further supported the performance of this model (Figure S2).

In an alternative analysis, we estimated a model that distinguished between glomerular filtration (fixed to 7.2 L/h for a subject with normal body size) and renal tubular secretion clearance. For this alternative model, glomerular filtration was affected by protein binding, whereas tubular secretion was not. The estimated differences in protein binding were well comparable between this alternative model (Table S2) and the final model (Table 4). Both models had adequate predictive performance. While these models are not nested, the −2x log-likelihood was better by 7.1 for the final (Table 4) compared to the alternative model (Table S2).

**Figure 4:**
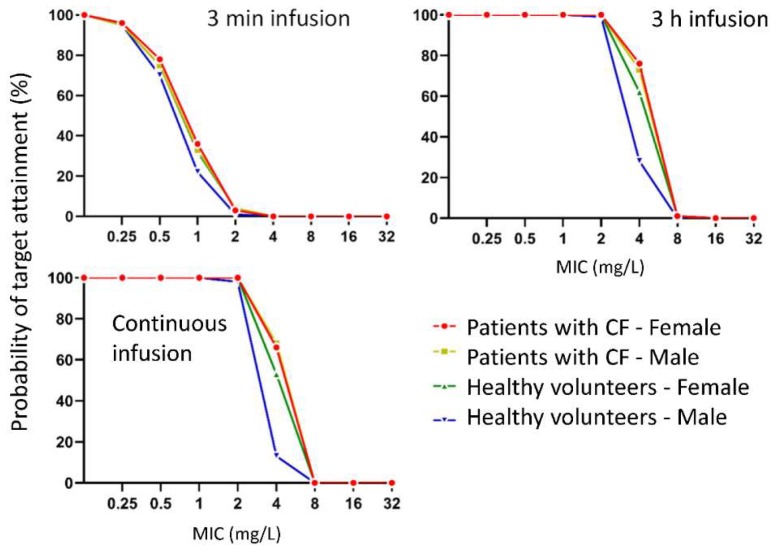
Probability of target attainment plot over a range of minimal inhibitory concentrations (MICs) for the bacteriostasis targets (40% *f*T_>MIC_) in female and male patients with CF and healthy volunteers. A probability of target attainment of 90% was defined as the pharmacokinetic/pharmacodynamic (PK/PD) breakpoint.

The simulated probabilities of target attainment were near-identical in female and male patients with CF (Figure 4) since both sexes were simulated with the same geometric mean LBM. Female healthy volunteers had marginally higher probabilities of target attainment than male healthy volunteers since females were smaller and the dose (3000 mg cefotiam per day) was not LBM-adjusted (Figure 4). Cefotiam short-term (3 min) infusions of 1000 mg every 8 h achieved robust (> 90%) probabilities of target attainment for the bacteriostasis target (40% *f*T_>MIC_) up to an MIC of 0.25 mg/L for all four subject groups (Table 5). Both 3 h prolonged infusions of 1000 mg every 8 h and the continuous infusion of 3000 mg/day achieved robust probabilities of target attainment up to an MIC of 2 mg/L. For the near-maximal bacterial killing target of 65% *f*T_>MIC_, continuous infusion was predicted to cover isolates with an MIC of up to 2 mg/L. However, prolonged infusions only covered MICs of 0.25 mg/L and short-term infusions up to 0.0625 mg/L (Table 5).

## 4. Discussion

While β-lactams are relatively hydrophilic molecules [39,40] with a protein binding of 40% or less in human plasma for many compounds, several β-lactams, especially those with Gram-positive activity, display a higher protein binding [3,5,6,16,41,42,43]. These more highly bound β-lactams include dicloxacillin, cloxacillin, methicillin, ticarcillin and aztreonam of which PK has been compared between patients with CF and healthy volunteers [5,6,9,44,45,46]. In these six studies, the ratio of the clearance in patients with CF divided by the clearance in healthy volunteers was 1.68 ± 0.67 (average ± SD) when calculated based on total plasma concentrations; after accounting for the higher unbound fractions in patients with CF relative to those in healthy volunteers, this ratio became 1.32 ± 0.42 for unbound clearances. As reviewed recently [3], some of these remaining PK differences could have been caused by adolescent and presumably rather morbid patients with CF being compared to adult healthy volunteers in these early PK studies. Hypoalbuminemia has been observed in patients with CF [2,4] and may lead to a lower plasma protein binding. Moreover, hypoalbuminemia is an independent risk factor for death in patients with CF and other conditions after lung transplantation [47].

Cefotiam is a cephalosporin with a protein binding of 40% to 62% in human plasma [15,16,17,18]. We are not aware of studies on the PK or protein binding of cefotiam in patients with CF. While we did not quantify protein binding in this study, our models considered that cefotiam may have a larger unbound fraction in plasma of patients with CF compared to that in healthy volunteers based on results on other β-lactams [5,6,9,44,45,46,48]. Our non-compartmental PK parameter estimates (Table 2) were based on total cefotiam concentrations and were in good agreement with previous reports for cefotiam in healthy volunteers [49,50,51,52]. After accounting for the differences in body size and body composition via allometric scaling by LBM via population PK modeling, patients with CF still had 11% to 38% larger clearances and volumes of distribution compared to those in healthy volunteers (Table 3).

Supported by the larger unbound fractions for relatively highly protein bound β-lactams in patients with CF compared to the unbound fractions in healthy volunteers [3], we developed a novel population PK model. After accounting for body size and composition, this model explained the remaining PK differences by estimating the unbound fraction of cefotiam in plasma. We estimated higher unbound fractions in patients with CF compared to those in healthy volunteers and higher unbound fractions in females compared to those in males (Table 4). The latter result was supported by female patients with CF having a similar LBM (Table 1), yet consistently larger renal, non-renal and total clearances of cefotiam compared to those in male patients with CF (Table 2). Estimating different unbound fractions avoided the use of disease specific scale factors (Table 3) and simplified the model (i.e., required fewer parameters to be estimated). Scale factors were required for a model that used the same unbound fraction in all subject groups (Table S1). Thereby, estimating protein binding differences sought to explain the remaining PK alterations between patients with CF and healthy volunteers after accounting for the differences in body size and composition.

We considered an alternative model where renal clearance was split into a glomerular filtration and tubular secretion clearance similar to prior analyses [53,54,55,56]. Cefotiam is a low renal extraction ratio drug since its unbound renal clearance of 23.8 L/h is equivalent to approximately 32% of renal blood flow (74 L/h) [57]. In this alternative model, protein binding was modeled to affect only glomerular filtration. This model yielded well comparable parameter estimates (Table S2) and diagnostic plots compared to those of the final model (Table 4); however, the alternative model was more complex and prior studies showed that plasma protein binding affects renal transport of organic anions [58]. Likewise, protein binding also affects the active transport and secretion of cefonicid, a cephalosporin comparable to cefotiam, in isolated perfused rat kidneys [59]. This suggested that the entire renal clearance was affected by protein binding as implemented in the final model (Table 4). We chose not to include a potential covariate effect for glomerular filtration rate on renal clearance to keep the model slightly simpler.

Limitations of our study include that we could not determine the unbound fraction of cefotiam in our subjects since plasma samples were no longer available at the time of this modeling. Additional limitations include the small sample size of this study and that we did not have data on albumin concentrations in our subjects; thus, the effect of albumin on protein binding was not included in the model. However, our results were in good agreement with the reported protein binding and PK differences between patients with CF and healthy volunteers for aztreonam [9] and for other moderately or highly protein bound β-lactams [5,6,9,44,45,46]. Significantly lower serum albumin and prealbumin concentrations have been reported for patients with CF compared to control subjects [60]. Moreover, significantly lower albumin concentrations were found in female (*n* = 187) compared to male (*n* = 306) patients with CF who had severe liver disease, pancreatic insufficiency and portal hypertension [61]. The latter patients with CF were more morbid than those in our study. However, both of these studies [60,61] support our modeling results with higher unbound fractions for cefotiam in female and male patients with CF compared to those in healthy volunteers (see also Supplementary Materials).

Cefotiam is used for the treatment of intra-abdominal infections [19] and for antibiotic prophylaxis of intra-abdominal, urological, biliary and other surgeries [62,63,64,65,66,67,68] in some countries. For our Monte Carlo situations, we considered the bacteriostasis target of 40% *f*T_>MIC_. We simulated male and female patients with CF with the same body size (i.e., mean LBM of 40 kg) and found near-identical probabilities of target attainment in both sexes for a non-size-adjusted dose of 3000 mg per day (Figure 4). This was expected since the unbound clearances and unbound volumes of distribution were the same in all subject groups when subjects had the same body size (Table 4). This was in agreement with a previous study on aztreonam which reported a similar unbound clearance for patients with CF and healthy volunteers [9]. While the higher unbound fractions in female patients with CF, for example, affected the total cefotiam concentrations in plasma, the difference in protein binding had no impact on the unbound concentrations. Therefore, the PK/PD breakpoints were identical in female and male patients with CF. For a fixed dose of 3000 mg cefotiam per day, female healthy volunteers achieved slightly higher probabilities of target attainment since they were smaller than male healthy volunteers (Figure 4). Our PK/PD breakpoints in patients with CF and healthy volunteers were in agreement with those from one previous study on cefotiam in patients with intra-abdominal infections [19]. Overall, these results provide guidance to clinicians about the benefit of prolonged compared to short-term infusions of cefotiam.

## 5. Conclusions

This study was the first to compare the population PK of cefotiam in patients with CF to that in healthy volunteers. We accounted for the differences in body size and body composition between both subject groups via allometric scaling by LBM. After accounting for body size and composition, renal clearance was 21% larger, non-renal clearance was 11% larger and volume of distribution was 38% larger in patients with CF compared to those in healthy volunteers when calculating these PK parameters based on total drug concentrations. Within the perspective of literature data on the PK of β-lactams with moderate or high protein binding in patients with CF, these PK differences were expected for cefotiam which has an unbound fraction in plasma of approximately 50% in healthy volunteers. Our final population PK model explained the PK differences by estimating higher unbound fractions of 74.4% in female and 56.3% in male patients with CF compared to 54.5% in female and 50% in male healthy volunteers. For female and male patients with CF who had the same body size, a fixed dose of 3000 mg cefotiam per day yielded identical probabilities of target attainment and PK/PD breakpoints in both sexes. Prolonged and continuous infusions achieved 8-fold higher PK/PD breakpoints than short-term infusions every 8 h for the bacteriostasis target. Overall, the proposed novel population modeling approach holds promise to describe potential PK differences in special patient populations with altered protein binding.

## Figures and Tables

**Table 1:** Demographic data of patients with cystic fibrosis (CF) and healthy volunteers (data are median [range]).

Demographic Variable	Patients with CF	Healthy Volunteers
Number of subjects (males/females)	8 (4/4)	6 (3/3)
Age (yr)	19 [17–24]	23.5 [21–26]
Height (cm)	167 [157–173]	169 [164–190]
Total body weight (WT) (kg)	45.5 [33.0–59.0]	68.5 [58.0–80.0]
WT in females	48.0 [33.0–59.0]	58.0 [58.0–62.0]
WT in males	44.9 [44.6–53.5]	80.0 [75.0–80.0]
Lean body mass (LBM) a (kg)	40.3 [28.8–46.2]	50.6 [44.6–65.4]
LBM in females	38.8 [28.8–45.7]	44.6 [44.6–45.4]
LBM in males	40.4 [39.6–46.2]	62.8 [55.8–65.4]
Body mass index (kg m^−2^)	17.0 [13.4–19.9]	22.5 [20.3–27.9]

^a^: Calculated based on the formula by Cheymol and James [33,34].

**Table 2:** Unscaled pharmacokinetic (PK) parameters from non-compartmental analysis (data are median [range]). These PK parameters were calculated based on total cefotiam concentrations in plasma.

Pharmacokinetic Parameter	Patients with CF	Healthy Volunteers
Total clearance (L/h)	17.1 [8.97–27.8]	17.7 [16.2–24.0]
in females	22.1 [15.0–27.8] ^a^	16.2 [16.2–18.5] ^a^
in males	15.9 [8.97–21.1] ^a^	19.1 [16.9 - 24.0] ^a^
Renal clearance (L/h)	12.0 [4.27–19.5]	11.6 [10.6–17.0]
in females	15.5 [10.7–19.5] ^a^	11.8 [10.6–12.5] ^a^
in males	11.7 [4.27–12.6] ^a^	11.3 [10.9–17.0] ^a^
Non-renal clearance (L/h)	5.08 [3.19–8.47]	5.97 [4.41–7.76]
in females	6.52 [4.22–8.26] ^a^	5.65 [4.41–6.01] ^a^
in males	4.96 [3.19–8.47] ^a^	7.04 [5.93–7.76] ^a^
Volume of distribution at steady-state (L)	12.4 [8.80–18.1]	12.8 [10.5–16.7]
in females	13.3 [8.80–18.1] ^a^	10.7 [10.5–13.3] ^a^
in males	12.3 [10.6–13.6] ^a^	16.7 [12.4–16.7] ^a^
Peak concentration (mg/L)	124 [74.1–293]	111 [81.7–130]
Terminal half-life (h)	0.931 [0.881–1.91]	1.08 [0.753–1.66]
Mean residence time (h)	0.699 [0.527–1.22]	0.707 [0.646–0.874]
Fraction of dose excreted unchanged into urine (%)	70.3% [47.6–77.8%]	66.3% [59.4–72.7%]

^a^: Female and male patients with CF had a similar median LBM (4% larger in males). However, LBM was 41% larger in male compared to female healthy volunteers.

**Table 3:** Disease factors representing the group estimate in patients with CF divided by the group estimate in healthy volunteers for the respective clearance or volume of distribution parameters.

Body Size Model ^a^	FCYF_CLr_	FCYF_CLnr_	FCYF_Vss_
1) No body size model	0.99 (22.7%)	0.90 (11.4%)	1.03 (53.1%)
2) WT linear scaling	1.43 (12.4%)	1.31 (22.7%)	1.52 (12.4%)
3) WT allometric	1.31 (10.4%)	1.19 (7.7%)	1.52 (14.0%)
4) LBM linear scaling	1.29 (12.4%)	1.19 (22.9%)	1.38 (14.8%)
5) LBM allometric	1.21 (7.5%)	1.11 (14.7%)	1.38 (6.9%)

^a^: This table compares the results for the different body size models for subjects of standard body size (i.e., a WT_STD_ of 70 kg or LBM_STD_ of 53 kg). An ideal body size model should explain the differences in body size and body composition and thus yield disease specific scale factors close to 1.0.

**Table 4:** Population pharmacokinetic parameter estimates for unbound cefotiam in patients with CF and healthy volunteers. All parameter estimates (except the additive residual errors) refer to unbound cefotiam. The model used allometric scaling with a standard LBM_STD_ of 53 kg.

Pharmacokinetic Parameter	Symbol	Unit	Population Mean (SE%)	BSV ^a^ (SE%)
Unbound renal clearance	CLr_u_	L/h	23.8 (6.9%)	0.237 (52.7%)
Unbound nonrenal clearance	CLnr_u_	L/h	11.0 (7.0%)	0.237 (50.2%)
Unbound total clearance	CLtot_u_	L/h	34.8 ^b^	
Unbound volume of distribution of central compartment	V1_u_	L	15.6 (6.5%)	0.189 (74.0%)
Unbound volume of distribution of shallow peripheral compartment	V2_u_	L	6.91 (14.1%)	0.256 (88.2%)
Unbound volume of distribution of deep peripheral compartment	V3_u_	L	4.56 (16.4%)	0.451 (131%)
Unbound volume of distribution at steady-state	Vss_u_	L	27.1 ^c^	
Unbound distribution clearance for shallow peripheral compartment	CLd_shallow,u_	L/h	13.8 (15.0%)	0.416 (183%)
Unbound distribution clearance for deep peripheral compartment	CLd_deep,u_	L/h	1.84 (26.1%)	0.309 (83.8%)
Unbound fraction in plasma for females with CF	fu_CF,F_		0.744 (4.5%) ^d^	
Unbound fraction in plasma for males with CF	fu_CF,M_		0.563 (13.5%) ^d^	
Unbound fraction in plasma for female healthy volunteers	fu_HV,F_		0.545 (13.6%) ^d^	
Unbound fraction in plasma for male healthy volunteers	fu_HV,M_		0.50 (fixed)	
SD of additive residual error for plasma concentrations	SDin	mg/L	0.0186 (53.7%)	
Proportional residual error for plasma concentrations	SDsl		0.166 (7.8%)	
SD of additive residual error for fraction of dose in urine	UDin	%	0.384 (76.1%)	

^a^: Between subject variability reported as apparent coefficient of variation of a normal distribution on natural logarithmic scale. The relative standard errors (SE%) refer to the estimated variances. ^b^: Calculated based on the estimated renal and nonrenal clearances. ^c^: Calculated as the sum of the three estimated volumes of distribution. ^d^: Unbound fraction was fixed to 0.5 in male healthy volunteers based on literature data. The population means of the remaining three unbound fractions were estimated separately for males and females with a small fixed between subject variability (5% coefficient of variation).

**Table 5:** PK/PD breakpoints (i.e., the highest MICs [in mg/L] with a probability of target attainment of at least 90%) for three cefotiam dosage regimens in patients with CF and healthy volunteers. All simulated regimens used a daily dose of 3000 mg cefotiam (not adjusted by body weight).

**Dosage Regimen**	**Bacteriostasis Target (40% *f*T_>MIC_)**			
	**Patients with CF**		**Healthy Volunteers**	
	**Female**	**Male**	**Female**	**Male**
Continuous infusion of 3000 mg/day (with a 500 mg loading dose at 0 h)	2	2	2	2
Prolonged (3 h) infusions of 1000 mg every 8 h	2	2	2	2
Short-term (3 min) infusions of 1000 mg every 8 h	0.25	0.25	0.25	0.25
	**Near-Maximal Killing Target (65% *f*T_>MIC_)**			
Continuous infusion of 3000 mg/day (with a 500 mg loading dose at 0 h)	2	2	2	2
Prolonged (3 h) infusions of 1000 mg every 8 h	0.25	0.25	0.25	0.25
Short-term (3 min) infusions of 1000 mg every 8 h	0.0625	0.0625	0.0625	0.0625

## Supplementary Materials

The following are available online at https://www.mdpi.com/1999-4923/11/6/286/s1, Figure S1. Plasma concentrations of cefotiam after a single 3 min intravenous infusion of 1027.5 mg in patients with CF and healthy volunteers. Figure S2. Observed and individual (left) or population (right) fitted total plasma concentrations of cefotiam on linear (top) and logarithmic (bottom) scale. Table S1. Population pharmacokinetic parameter estimates for unbound cefotiam in patients with cystic fibrosis and healthy volunteers for the model that included disease specific scaling factors. All parameter estimates (except the additive residual errors) refer to unbound cefotiam; for this model, the unbound fraction was fixed to 0.5 in all subject groups based on literature data. This model used allometric scaling by LBM with an LBM_STD_ of 53 kg. Table S2. Population pharmacokinetic parameter estimates for unbound cefotiam in patients with CF and healthy volunteers for the alternative model which distinguished between glomerular filtration and renal tubular secretion clearance; the latter was not affected by protein binding. All parameter estimates (except the additive residual errors) refer to unbound cefotiam. The model used allometric scaling with a standard LBM_STD_ of 53 kg.
