# Supplementary material for: Novel Population Pharmacokinetic Approach to Explain the Differences between Cystic Fibrosis Patients and Healthy Volunteers via Protein Binding
Source: Pharmaceutics. 2019 Jun 18;11(6):286. doi: 10.3390/pharmaceutics11060286 (PMC6630667; doi:10.3390/pharmaceutics11060286)
Supplement: Supplementary file 1 [file pharmaceutics-11-00286-s001.pdf]

pharmaceutics

Supplementary Materials: Novel Population Pharmacokinetic Approach to Explain the Differences between Cystic Fibrosis Patients and Healthy Volunteers via Protein Binding

Nirav R. Shah, Jürgen B. Bulitta, Martina Kinzig, Cornelia B. Landersdorfer, Yuanyuan Jiao, Dhruvitkumar S. Sutaria, Xun Tao, Rainer Höhl, Ulrike Holzgrabe, Frieder Kees, Ulrich Stephan and Fritz Sörgel

Relationship between Albumin Concentrations and Protein Binding

We used literature data on the differences in albumin concentrations between patients with cystic fibrosis (CF) and healthy volunteers to explore whether the estimated differences in unbound fractions (fu) in our subjects were physiologically plausible. Based on our modeled fu estimates, we back-calculated the ratio of albumin concentrations between patients with CF and healthy volunteers. This ratio was compared to literature data on albumin concentrations in both subject groups.

We assumed that the binding affinity of cefotiam to albumin was the same between patients with CF and healthy volunteers. The fu then depends on the albumin concentration. For the plasma protein binding assay, diluted plasma is sometimes used to determine fu. We employed the standard equation for converting fu in diluted plasma to fu in undiluted plasma [1] to predict the impact of different albumin concentrations between patients with CF and healthy volunteers. This equation was rearranged to calculate the ratio (R) of albumin concentrations in patients with CF divided by that in healthy volunteers. This yielded the following equation:

$$\mathrm{fu(HV)} = \left(\frac{\mathrm{fu(CF)}}{\mathrm{R - (R\text{-}1)\ \cdot fu(CF)}} \right)$$

where the fu(HV) is the unbound fraction in healthy volunteers and fu(CF) the unbound fraction in patients with CF. When inserting the estimates for female healthy volunteers [fu(HV) = 0.545] and female patients with CF [fu(CF) = 0.744], this yielded a 2.42-fold higher albumin concentration in female healthy volunteers compared to female patients with CF. For males, albumin concentrations were predicted to be 1.39-fold higher in healthy volunteers compared to patients with CF.

Plasma albumin concentrations of approximately 2.49 g/dL were reported for patients with CF, depending on their degree of morbidity [2–5]. The normal range of albumin concentrations in healthy volunteers is 3.4–5.4 g/dL. This yields a range from 1.37 to 2.17 for the ratio of albumin concentrations in healthy volunteers divided by that in patients with CF. This range was in good agreement with the back-calculated ratios (1.39 and 2.42) based on the estimated unbound fractions from our population PK modeling. Thus, the estimated differences in the unbound fraction were physiologically plausible.

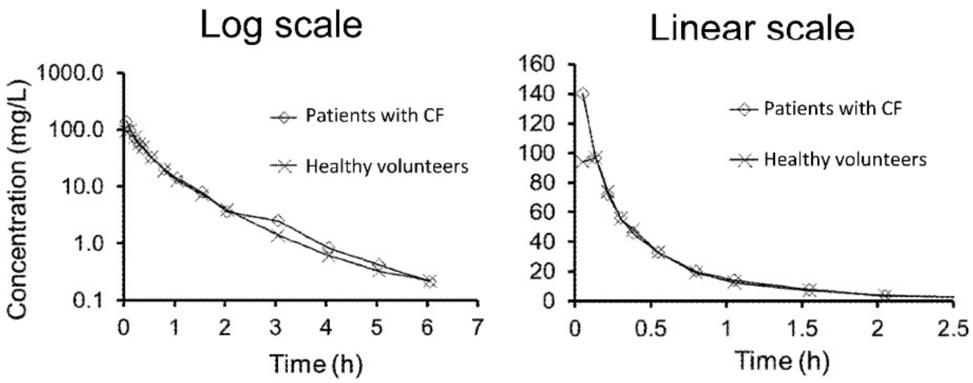

Figure S1. Plasma concentrations of cefotiam after a single 3 min intravenous infusion of 1027.5 mg in patients with CF and healthy volunteers.

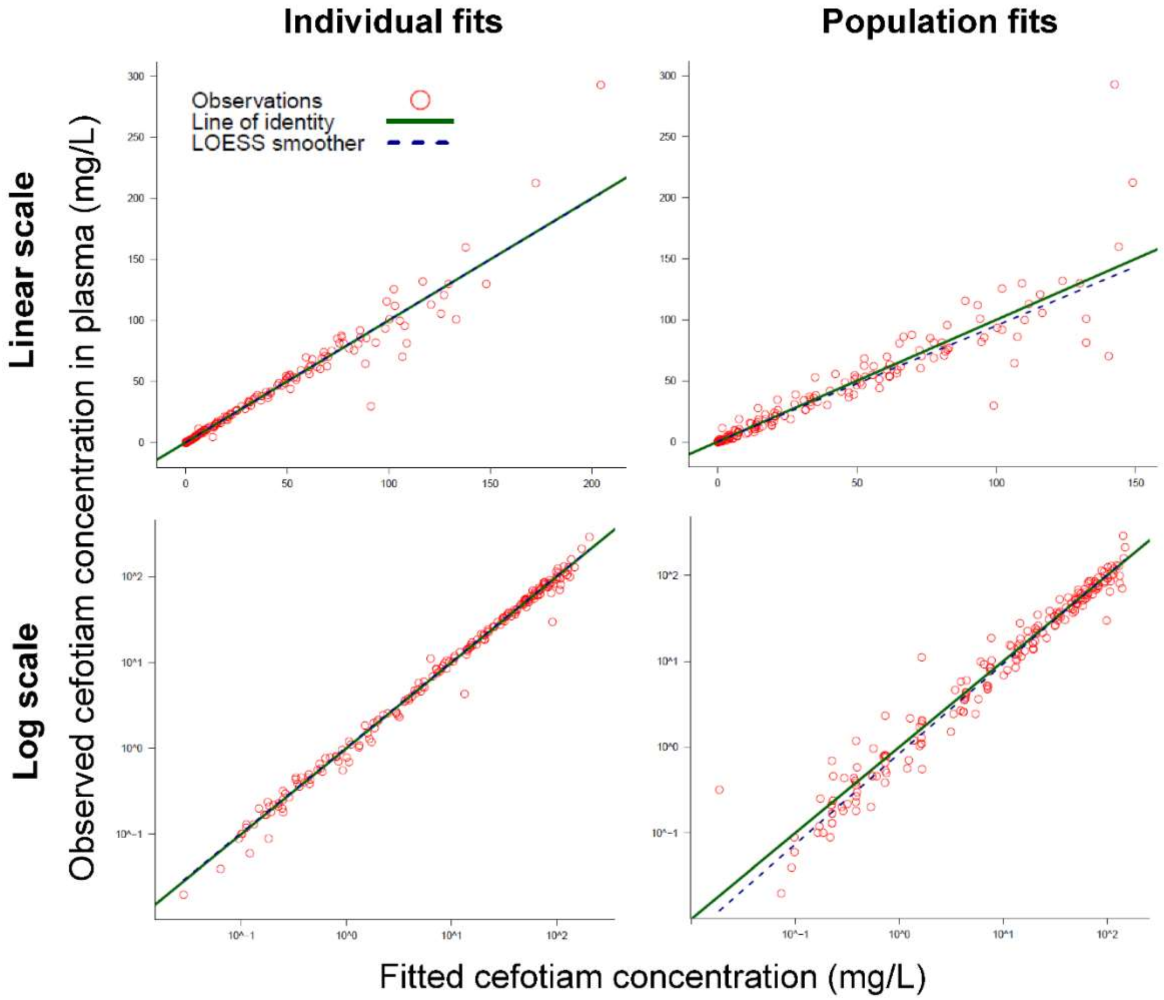

Figure S2. Observed and individual (left) or population (right) fitted total plasma concentrations of cefotiam on linear (top) and logarithmic (bottom) scale.

Table S1. Population pharmacokinetic parameter estimates for unbound cefotiam in patients with cystic fibrosis and healthy volunteers for the model that included disease specific scaling factors. All parameter estimates (except the additive residual errors) refer to unbound cefotiam; for this model, the unbound fraction was fixed to 0.5 in all subject groups based on literature data. This model used allometric scaling by LBM with an LBM_{STD} of 53 kg.

Pharmacokinetic Parameter	Symbol	Unit	Patients with cystic fibrosis	Healthy volunteers	Population mean (SE%)	BSV[a] (SE%)
			Population mean (SE%)	BSV[a] (SE%)		
Unbound renal clearance	CLr_u	L/h	30.3 (11.53%)	0.101 (45.1%)	25.0 (8.76%)	0.101 (45.1%)
Unbound nonrenal clearance	$CLnr_u$	L/h	13.6 (17.67%)	0.065 (47.9%)	12.2 (9.81%)	0.065 (47.9%)
Unbound total clearance	$CLtot_u$	L/h	43.9 [b] (6.76%)		37.2 [b] (6.70%)	
Unbound volume of distribution of central compartment	$V1_u$	L	21.25 (10.84%)	0.062 (48.4%)	15.4 (8.37%)	0.062 (48.4%)
Unbound volume of distribution of shallow peripheral compartment	$V2_u$	L	10.5 (10.73%)	0.015 (140%)	7.61 (8.23%)	0.015 (140%)
Unbound volume of distribution of deep peripheral compartment	$V3_u$	L	5.74 (23.24%)	0.277 (80.9%)	4.17 (22.2%)	0.277 (80.9%)
Unbound volume of distribution at steady-state	Vss_u	L	37.50 [c] (7.71%)		27.2 [c] (6.27%)	
Unbound distribution clearance for shallow peripheral comp.	$CLd_{shallow,u}$	L/h	15.9 (20.7%)	0.161 (60.2%)	15.9 (20.7%)	0.161 (60.2%)
Unbound distribution clearance for deep peripheral comp.	$CLd_{deep,u}$	L/h	1.47 (19.1%)	0.0454 (159%)	1.47 (19.1%)	0.0454 (159%)
SD of additive residual error for plasma concentrations	SDin	mg/L	0.0217 (47.9%)		0.0217 (47.9%)	
Proportional residual error for plasma concentrations	SDsl		0.165 (7.42%)		0.165 (7.42%)	
SD of additive residual error for fraction of dose in urine	UDin	%	0.529 (85.6%)		0.529 (85.6%)	

[a]: Between subject variability reported as apparent coefficient of variation of a normal distribution on natural logarithmic scale. The relative standard errors (SE%) refer to the estimated variances. [b]: Calculated based on the estimated renal and nonrenal clearances (i.e. not an estimated parameter). [c]: Calculated as the sum of the three estimated volumes of distribution (i.e. not an estimated parameter).

Table S2. Population pharmacokinetic parameter estimates for unbound cefotiam in patients with CF and healthy volunteers for the alternative model which distinguished between glomerular filtration and renal tubular secretion clearance; the latter was not affected by protein binding. All parameter estimates (except the additive residual errors) refer to unbound cefotiam. The model used allometric scaling with a standard LBM_{STD} of 53 kg.

Pharmacokinetic Parameter	Symbol	Unit	Population mean (SE%)	BSV [a] (SE%)
Unbound glomerular filtration clearance	$CLr_{GFR,u}$	L/h	7.20 (fixed)	0 (not estimated)
Renal tubular secretion (not affected by protein binding)	CLr_{SEC}	L/h	9.64 (12.4%)	0.457 (43.0%)
Unbound nonrenal clearance	$CLnr_u$	L/h	11.1 (7.9%)	0.244 (56.8%)
Unbound volume of distribution of central compartment	$V1_u$	L	15.9 (6.5%)	0.191 (54.4%)
Unbound volume of distribution of shallow peripheral compartment	$V2_u$	L	7.10 (11.7%)	0.234 (65.8%)
Unbound volume of distribution of deep peripheral compartment	$V3_u$	L	4.59 (20.1%)	0.468 (66.9%)
Unbound volume of distribution at steady-state	Vss_u	L	27.6 [c]	
Unbound distribution clearance for shallow peripheral compartment	$CLd_{shallow,u}$	L/h	14.2 (18.1%)	0.380 (62.9%)
Unbound distribution clearance for deep peripheral compartment	$CLd_{deep,u}$	L/h	1.78 (30.8%)	0.339 (90.6%)
Unbound fraction in plasma for females with CF	$fu_{CF,F}$		0.717 (16.0%) [d]	
Unbound fraction in plasma for males with CF	$fu_{CF,M}$		0.561 (11.1%) [d]	
Unbound fraction in plasma for female healthy volunteers	$fu_{HV,F}$		0.533 (10.4%) [d]	
Unbound fraction in plasma for male healthy volunteers	$fu_{HV,M}$		0.500 (fixed)	
SD of additive residual error for plasma concentrations	SDin	mg/L	0.0191 (50.6%)	
Proportional residual error for plasma concentrations	SDsl		0.165 (7.8%)	
SD of additive residual error for fraction of dose in urine	UDin	%	0.190 (98.7%)	

[a]: Between subject variability reported as apparent coefficient of variation of a normal distribution on natural logarithmic scale. The relative standard errors (SE%) refer to the estimated variances. [b]: Calculated based on the estimated renal and nonrenal clearances. [c]: Calculated as the sum of the three estimated volumes of distribution. [d]: Unbound fraction was fixed to 0.5 in male healthy volunteers based on literature data. The population means of the remaining three unbound fractions were estimated separately for males and females with a small fixed between subject variability (5% coefficient of variation).

Berkeley Madonna Monte Carlo simulation code for cefotiam

```
;###########################################################################
; Differential equation settings

Method STIFF
STARTTIME = 0 ; time when the simulation starts
STOPTIME   = 32 ; time when the simulation ends
DT         =1e-3 ; Integration time step (only used for RK4)
DTMIN     =1e-5 ; minimum time step of the Auto and Stiff diff. equation solvers
DTMAX     =0.01 ; minimum time step of the Auto and Stiff diff. equation solvers
TOLERANCE =0.00001 ; relative accuracy for Auto and Stiff diff. eqn. solver
DTOUT     = 0.05 ; Output a simulation point ever nn time units


LBM      = 53 ; kg
GRP      = 1 ;
SEX1F_0M = 1 ;

LBM_Mean_CFM = 40; kg
LBM_CV_CFM   =  0.15 ; fixed

LBM_Mean_CFF = 40; kg
LBM_CV_CFF   =  0.15 ; fixed

LBM_Mean_HVM = 61.33 ; kg
LBM_CV_HVM  =  0.15 ; fixed

LBM_Mean_HVF = 44.90 ; kg
LBM_CV_HVF  =  0.15 ; fixed


LBM_Mean = IF (GRP = 1)  THEN   IF (SEX1F_0M = 0) THEN LBM_Mean_CFM  ELSE  LBM_Mean_CFF  ELSE
                               IF (SEX1F_0M = 0) THEN LBM_Mean_HVM  ELSE  LBM_Mean_HVF

LBM_CV   = IF (GRP = 1)  THEN   IF (SEX1F_0M = 0) THEN LBM_CV_CFM     ELSE  LBM_CV_CFF    ELSE
                               IF (SEX1F_0M = 0) THEN LBM_CV_HVM    ELSE  LBM_CV_HVF

; Log normal distribution for LBM
init ETA_LBM = normal(0, LBM_CV)
next ETA_LBM = ETA_LBM
LBM          = LBM_Mean * EXP(ETA_LBM)



;$DIFFEQ_DIF

DC1 = Cent/V1cov ; Conc. of cefotiam in central compartment
DC2 = Shal/V2cov ; Conc. of cefotiam in shallow peripheral compartment
DC3 = Deep/V3cov ; Conc. of cefotiam in deep peripheral compartment

; Dosing of Cefotiam
Dur_min = 3 ; min
Dur     = Dur_min / 60
Tau     = 8 ; h

Dose    = 1000 ; mg

DurBol   = 0.05 ; h
DoseBol  = 1000 ; mg

InfBolus = DoseBol/DurBol * squarepulse(0,DurBol)

Inf1 = Dose/Dur * squarepulse(0*Tau,Dur)
Inf2 = Dose/Dur * squarepulse(1*Tau,Dur)
Inf3 = Dose/Dur * squarepulse(2*Tau,Dur)
Inf4 = Dose/Dur * squarepulse(3*Tau,Dur)
Inf5 = Dose/Dur * squarepulse(4*Tau,Dur)
Inf6 = Dose/Dur * squarepulse(5*Tau,Dur)

INPUT = InfBolus + Inf1 + Inf2 + Inf3 + Inf4 + Inf5 + Inf6

d/dt(Cent)    = INPUT-CLRcov*DC1-CLNRcov*DC1 -CLDcov*DC1+CLDcov*DC2 -CLD3cov*DC1+CLD3cov*DC3
d/dt(Shal)    =                              CLDcov*DC1-CLDcov*DC2
d/dt(Deep)    =                                                      CLD3cov*DC1-CLD3cov*DC3
d/dt(Urin)    =       CLRcov*DC1
```

```
init AUC_Cent   = 0
init AUC_Shal   = 0
init AUC_Deep   = 0
init CUM_Dose   = 0

d/dt(AUC_Cent) = DC1
d/dt(AUC_Shal) = DC2
d/dt(AUC_Deep) = DC3
d/dt(CUM_Dose) = INPUT


;$OUTPUT_GLB

FU  = IF (GRP = 1)  THEN   IF (SEX1F_0M = 0) THEN FU_CFM ELSE FU_CFF ELSE
                           IF (SEX1F_0M = 0) THEN FU_HVM ELSE FU_HVF

; Allometric scaling equations for covariate effect
FWTCL = (LBM/53)**0.75
FWTV  = (LBM/53)

CLRcov  = FWTCL * CLR
CLNRcov = FWTCL * CLNR
CLDcov  = FWTCL * CLD
CLD3cov = FWTCL * CLD3

V1cov   = FWTV  * V1
V2cov   = FWTV  * V2
V3cov   = FWTV  * V3

C1  = Cent /V1cov / FU      ; total drug plasma concentrations of cefotiam (mg/L)

; Initial conditions
init Cent       = 0
init Shal       = 0
init Deep       = 0
init Urin       = 0


;############################################################################
; Parameter mean estimates and covariate effects (FIXED EFFECTS)

Mean_CLR        = 23.7911   ; L/h
Mean_CLNR   = 10.9943   ; L/h
Mean_CLD    = 13.8043   ; L/h
Mean_CLD3   = 1.83622   ; L/h
Mean_V1     = 15.5927   ; L
Mean_V2     = 6.90932   ; L
Mean_V3     = 4.5646    ; L
Mean_FU_CFF = 0.74359   ; -
Mean_FU_CFM = 0.562527  ; -
Mean_FU_HVF = 0.544842  ; -
FU_HVM      = 0.5       ; -


;############################################################################
; Parameter variability estimates (coefficients of variation or SDs)

CV_CLR      = 0.236551  ;
CV_CLNR     = 0.237031  ;
CV_CLD      = 0.415826  ;
CV_CLD3     = 0.308592  ;
CV_V1       = 0.189451  ;
CV_V2       = 0.256252  ;
CV_V3       = 0.450914  ;
CV_FU_CFF   = 0.05  ;
CV_FU_CFM   = 0.05  ;
CV_FU_HVF   = 0.05  ;
```

```
;########################################
;# Monte Carlo part:   Generation of random deviates

init ETA_CLR    = normal(0, CV_CLR)
init ETA_CLNR   = normal(0, CV_CLNR)
init ETA_CLD    = normal(0, CV_CLD)
init ETA_CLD3   = normal(0, CV_CLD3)
init ETA_V1     = normal(0, CV_V1)
init ETA_V2     = normal(0, CV_V2)
init ETA_V3     = normal(0, CV_V3)
init ETA_FU_CFF = normal(0, CV_FU_CFF)
init ETA_FU_CFM = normal(0, CV_FU_CFM)
init ETA_FU_HVF = normal(0, CV_FU_HVF)

next ETA_CLR    = ETA_CLR
next ETA_CLNR   = ETA_CLNR
next ETA_CLD    = ETA_CLD
next ETA_CLD3   = ETA_CLD3
next ETA_V1     = ETA_V1
next ETA_V2     = ETA_V2
next ETA_V3     = ETA_V3
next ETA_FU_CFF = ETA_FU_CFF
next ETA_FU_CFM = ETA_FU_CFM
next ETA_FU_HVF = ETA_FU_HVF

CLR          = Mean_CLR     * EXP(ETA_CLR)
CLNR         = Mean_CLNR    * EXP(ETA_CLNR)
CLD          = Mean_CLD     * EXP(ETA_CLD)
CLD3         = Mean_CLD3    * EXP(ETA_CLD3)
V1           = Mean_V1      * EXP(ETA_V1)
V2           = Mean_V2      * EXP(ETA_V2)
V3           = Mean_V3      * EXP(ETA_V3)
FU_CFF       = Mean_FU_CFF * EXP(ETA_FU_CFF)
FU_CFM       = Mean_FU_CFM * EXP(ETA_FU_CFM)
FU_HVF       = Mean_FU_HVF * EXP(ETA_FU_HVF)
```

References:

1. Riccardi, K.; Cawley, S.; Yates, P.D.; Chang, C.; Funk, C.; Niosi, M.; Lin, J.; Di, L. Plasma protein binding of challenging compounds. *J. Pharm. Sci.* **2015**, *104*, 2627–2636.
2. Baldwin, M.R.; Arcasoy, S.M.; Shah, A.; Schulze, P.C.; Sze, J.; Sonett, J.R.; Lederer, D.J. Hypoalbuminemia and early mortality after lung transplantation: A cohort study. *Am. J. Transplant.* **2012**, *12*, 1256–1267.
3. Stonebraker, J.R.; Ooi, C.Y.; Pace, R.G.; Corvol, H.; Knowles, M.R.; Durie, P.R.; Ling, S.C. Features of severe liver disease with portal hypertension in patients with cystic fibrosis. *Clin. Gastroenterol. Hepatol.* **2016**, *14*, 1207–1215 e1203.
4. Pittman, F.E.; Denning, C.R.; Barker, H.G. Albumin metabolism in cystic fibrosis. *Am. J. Dis. Child.* **1964**, *108*, 360–365.
5. Strober, W.; Peter, G.; Schwartz, R.H. Albumin metabolism in cystic fibrosis. *Pediatrics* **1969**, *43*, 416–426.
